# Prevalence and risk factors for persistent atrial fibrillation in obstructive sleep apnea

**DOI:** 10.5935/1984-0063.20220077

**Published:** 2022

**Authors:** Sittichai Khamsai, Chutimon Junkrasien, Panita Limpawattana, Jarin Chindaprasirt, Vichai Senthong, Watchara Boonsawat, Kittisak Sawanyawisuth

**Affiliations:** Khon Kaen University, Medicine - Khon Kaen - Thailand

**Keywords:** Prevalence, Age, Glomerular Filtration Rate, Obstructive Sleep Apnea

## Abstract

**Objectives:**

Obstructive sleep apnea (OSA) is a common cause of atrial fibrillation (AF). The prevalence rate of OSA in AF is highest at 80%. There is limited data if who will develop AF in OSA patients. This study aimed to evaluate the prevalence of AF in patients with OSA and find clinical factors predictive of AF in patients with OSA.

**Material and Methods:**

This was a cross-sectional study. We enrolled consecutive patients diagnosed with obstructive sleep apnea diagnosed by polysomnography. The primary outcome was persistent AF identified by electrocardiogram. Prevalence and predictors of AF in patients with OSA were analyzed.

**Results:**

During the study period, there were 199 patients with OSA enrolled in the study. Of those, 31 patients (15.57%) had AF. There were five factors in the final model predictive for AF in OSA patients. Among those factors, three factors were independently associated with AF in OSA including age, tiredness, and glomerular filtration rate. The latter two factors were protective factors, while age was a predictor for AF with an adjusted odds ratio (95% confidence interval) of 1.052 (1.004, 1.103).

**Conclusion:**

The prevalence of AF in patients with OSA was 15.57%. Elderly patients with renal deterioration are at risk of AF but AF risk was decreasing in patients with tiredness.

## INTRODUCTION

Atrial fibrillation (AF) is a common arrhythmia. It is reported that AF is associated with several cardiovascular conditions such as myocardial infarction, heart failure, and stroke^1^. The presence of AF may increase mortality by two times in patients with myocardial infarction^2^. There are several causes of AF such as mitral stenosis or obstructive sleep apnea (OSA).

OSA is prevalent and has a rising trend. A survey in the US found that 12-18 million adults may have untreated OSA which is associated with hypertension, stroke, and deaths^3-7^. Additionally, OSA is also associated with AF as recommended by the European Society of Cardiology guideline^8^. In nonvalvular AF, OSA is a common cause with a prevalence rate between 30-85%^9-12^.

Several mechanisms have been proposed for the association of OSA and AF including intermittent hypoxemia during sleep, intrathoracic pressure shifts, sympathovagal imbalance, or atrial remodeling^13^. Several studies also showed the risk factors of OSA in patients with AF^10-12^. The study from Poland found that risk factors of OSA in patients with AF were older (59.6 vs. 56.0 years; *p*<0.02), more obese (BMI=30.9 vs. 28.7kg/m^2^; *p*<0.01), and larger neck (41.2 vs. 39.3cm; *p*<0.01) than those without OSA^11^.

On the other hand, there is limited data on prevalence and risk factors of AF in patients with OSA^14,15^. A study from Germany found the prevalence of AF in OSA patients of 8.9%^14^. Patients with OSA tended to have older age (63.5 vs. 54.5 years; *p*<0.05) and had more proportions of hypertension and coronary artery diseases than those without OSA by unadjusted statistical method. This study aimed to evaluate the prevalence of AF in patients with OSA and find clinical factors predictive of AF in patients with OSA using the adjusted method. Additionally, this study was an additional study to pool up more data on this issue. Physicians may be able to predict AF occurrence in OSA suspected patients without a need for polysomnography.

## MATERIAL AND METHODS

This was a cross-sectional study conducted at Khon Kaen University’s obstructive sleep apnea clinic in Thailand. The study period was between September and December 2018. We enrolled all consecutive patients diagnosed with OSA by evidence of an apnea-hypopnea index (AHI) value of five events per hour or more by a polysomnography^16^. The primary outcome was persistent AF identified by electrocardiogram (ECG). Persistent AF was defined by the persistence of AF by resting electrocardiogram for more than seven days. AF has these three ECG characteristics including: 1) irregular R-R intervals (when atrioventricular conduction is present), 2) absence of distinct repeating P waves, and 3) irregular atrial activity^17^. Baseline characters, physical signs, and laboratory results of the eligible patients were studied. Risk factors, symptoms, and complications of OSA were also evaluated. For patients with AF, CHA2DS2VASc and HASBLED scores were evaluated to assess the risk of stroke and major bleeding risk from anticoagulants in patients with AF^18^.

### Statistical analysis

Eligible patients were divided by the presence of AF. Baseline and clinical characteristics of OSA patients with and without AF were compared by descriptive statistics. Multivariate logistic regression analysis was used to find factors predictive of AF in OSA. A univariate logistic regression analysis was performed to calculate the crude odds ratio (OR) of each studied factor. Factors with a *p*-value of <0.20 of crude odds ratios or clinical importance by previous research studies were put in the multivariate logistic regression analysis. Analytical results were presented as unadjusted/adjusted OR, and 95% confidence intervals. The goodness of fit of the final predictive model was computed by the Hosmer-Lemeshow method. All analyses were executed by STATA software, version 10.1 (College Station, TX, USA)

## RESULTS

During the study period, there were 199 OSA patients enrolled in the study. Of those, 31 patients (15.57%) had AF. There were six factors significantly different between AF and non-AF groups (Table 1) including age, sex, comorbid disease (stroke, heart failure, chronic kidney disease), and tiredness symptom. For example, the AF group had a significantly older age than the non-AF group (65 vs. 57 years; *p*<0.022) as shown in Table 1. Regarding physical signs and laboratory results, there was no significant difference between both groups (Tables 2 and 3). The AF group and nonAF group had comparable AHI (21 vs. 20 times/hour) as shown in Table 3. The AF group had CHA2DS2VASc and HASBLED score of 3 and 2, respectively.

There were five factors in the final model predictive for AF in patients with OSA (Table 4). Among those factors, three factors were independently associated with AF in patients with OSA including age, tiredness, and glomerular filtration rate. The latter two factors were protective factors, while age was a predictor of AF with an adjusted odds ratio (95% confidence interval) of 1.052 (1.004, 1.103). The Hosmer-Lemeshow chi-square of the predictive model was 9.84 (*p*<0.276).

**Table 1:** Baseline characteristics of obstructive sleep apnea (OSA) patients categorized by presence of atrial fibrillation (AF).

Factors	Non AF n=168	AF n=31	p-value
Median (1^st^-3^rd^ quartile range) age, years	57 (46-65)	65 (57-71)	0.022
Male sex, n (%)	109 (64.88)	11 (35.48)	0.003
Comorbid diseases			
Coronary artery disease	5 (3.40)	1 (3.23)	0.999
PVC	2 (1.36)	1 (3.23)	0.439
COPD	4 (2.72)	2 (6.43)	0.280
GERD	29 (19.73)	3 (9.68)	0.301
Diabetes mellitus	61 (41.50)	14(45.16)	0.842
Allergic rhinitis	33 (22.45)	7 (22.58)	0.999
Chronic kidney disease	45 (30.61)	3 (9.68)	0.015
Stroke	8 (5.44)	7 (22.58)	0.006
Hypertension	119 (80.95)	24 (77.42)	0.626
Heart failure	11 (7.48)	9 (29.03)	0.002
Hyperthyroidism	3 (8.11)	4 (20.00)	0.226
Tiredness	43 (87.76)	15 (57.69)	0.007
Excessive daytime sleepiness	68 (64.15)	16 (59.26)	0.660
Previous smoking	22 (22.92)	1 (8.33)	0.455
Previous alcohol	20 (21.51)	1 (9.09)	0.455
Current smoking	6 (6.19)	0	0.999
Current alcohol	5 (5.75)	1 (8.33)	0.549
Median (1^st^-3^rd^ quartile range) STOPBANG, points	5 (4-6)	5 (4-5)	0.587

Notes: Data presented as number (%) unless indicated otherwise; PVC = Premature ventricular contraction; COPD = Chronic obstructive pulmonary disease; GERD = Gastroesophageal reflux disease.

**Table 2:** Physical signs of obstructive sleep apnea patients categorized by presence of atrial fibrillation (AF).

Factors	Non AF n=168	AF n=31	p-value
Body mass index, kg/m^2^	29.3 (26.0-34.4)	29.0 (24.7-32.5)	0.322
Systolic blood pressure, mmHg	139 (128-150)	140 (128-155)	0.864
Diastolic blood pressure, mmHg	79 (71-85)	79 (70-87)	0.676
Neck circumference (cm)	41 (39-44)	43.5 (41-46.5)	0.163
Mallampati			0.573
Class I	12 (10.34)	3 (20.00)	
Class II	39 (33.62)	4(26.67)	
Class III	48 (41.38)	5 (33.33)	
Class IV	17 (14.66)	3 (20.00)	
Torus palatinus	13 (9.03)	2 (18.18)	0.288
Torus mandibularis	7 (4.90)	0	0.999
Tonsil enlargement	64 (38.10)	8 (25.81)	0.226
Microretrognathia	4 (2.74)	0	0.999
Macroglossia	47 (31.54)	4 (33.33)	0.999
Retrognathia	12 (8.39)	0	0.999

Notes: Data presented as median (1^st^-3^rd^ quartile range) or number (percentage).

**Table 3:** Laboratory results of obstructive sleep apnea patients categorized by presence of atrial fibrillation (AF).

Factors	Non AF n=168	AF n=31	p-value
Apnea hypopnea index (events/hr)	21 (11-42)	20 (12-28)	0.499
Fasting plasma glucose (mg/dL)	111 (95-131)	99 (96-116)	0.513
HbA1C (%)	6.2 (5.6-7.8)	6.3 (5.6-7.0)	0.884
Blood urea nitrogen (mg/dL)	14.8 (11.9-21.1)	17 (12.6-24.2)	0.300
Creatinine (mg/dL)	1.1 (0.8-1.4)	1.1 (0.8-1.3)	0.978
Glomerular filtration rate (ml/min)	73 (47-98)	65 (49-84)	0.131
Uric (mg/dL)	6.6 (5.4-7.6)	6.5 (5.3-9.4)	0.829
Albumin (g/dL)	4.2(3.8-4.5)	3.7 (3.4-4.1)	0.092
Cholesterol (mg/dL)	195 (160-221)	181 (140-220)	0.128
Triglyceride(mg/dL)	150 (96-192)	128 (97-162)	0.633
High-density lipoproteins (mg/dL)	48 (41-58)	49.5 (38.5-58.5)	0.933
Low-density lipoproteins (mg/dL)	127(95-159)	110 (85-159)	0.313

**Table 4:** Factors associated with occurrence of atrial fibrillation in obstructive sleep apnea patients.

Factors	Unadjusted odds ratio (95% confidence interval)	Adjusted odds ratio (95% confidence interval)
Age, years	1.028 (0.999, 1.058)	1.052 (1.004, 1.103)
Tiredness	0.190 (0.059, 0.604)	0.155 (0.032, 0.749)
Glomerular filtration rate	0.992 (0.979, 1.004)	0.967 (0.939, 0.996)
Body mass index	0.952 (0.891, 1.017)	1.026 (0.899, 1.169)
Daytime sleepiness	0.812 (0.342, 1.929)	0.444 (0.118, 1.665)

## DISCUSSION

The prevalence of atrial fibrillation in OSA patients was 15.57%. Predictors of AF in OSA patients included age, tiredness, and renal function.

As previously reported, increasing age was related to a higher incidence of AF^19^. The previous study showed that the age group of 85 years or over had an incidence rate of AF of 17.8%, while the AF incidence of the age group of 55-59 years was 0.7%^19^. In the older age group, predictors of AF were large body sizes which may be an indicator of OSA^20^. Additionally, OSA also has a higher prevalence in elderly patients with an increasing trend by age^21^. Therefore, it is not surprising that AF was increasing by age in OSA patients after adjusting for body mass index (Table 4). This finding was similar to the previous study from Germany even after adjusting for other variables in this study (Table 4)^22^.

Renal dysfunction had a higher prevalence of OSA and also a predictor of OSA in the elderly with an adjusted odds ratio of 2.32 (95%CI = 1.63, 3.31)^20^. Chronic kidney disease was reported to be a risk factor for new-onset AF (hazard ratio 4.65; 95%CI = 1.47, 14.70) by a study from Japan^22^. Therefore, increasing the glomerular filtration rate may reduce the risk of AF in OSA by 4% per 1ml/min/1.73m^2^ (Table 4).

Tiredness is one symptom listed in the STOP BANG questionnaire for OSA screening^23^. This symptom was very common and had a higher proportion than loud snoring in the STOP BANG questionnaire (48.0% vs. 41.8%)^24^. A previous study found that hypertension duration was a predictor of AF in obese OSA patients (adjusted odds ratio 1.10; 95%CI = 1.04, 1.16)^25^. These data may indicate that a longer duration of OSA is associated with an increasing risk of AF. As tiredness is the common symptom, OSA patients with tiredness may seek medical attention faster than those without tiredness resulting in a lower risk of repeated hypoxemia and AF occurrence (Table 4)^1^. In children with OSA, tiredness is also a common leading symptom (64%) presenting to the physicians^26^. Even though this study found that tiredness is negatively related to AF occurrence in patients with OSA, further studies are required to confirm the results of this study as well as the plausible mechanism of this association.

A recent review found that body mass index may be related to AF^23^. AF was contributed by body mass index in 17.9% of patients with AF. Additionally, obesity increases the risk of AF by 2.04 times^27^. A study from Nepal found that the rate of AF in patients with OSA was higher if body mass index of over 23.5kg/m^2^ compared to those with lower body mass index (6/7 vs. 1/7 patients)^15^. However, this study did not find a significant correlation between body mass index and AF in patients with OSA (Table 4). There are two possible explanations for these findings. First, our analysis was adjusted for other variables which are more robust and controlled for confounding factors. In other words, body mass index was not a strong predictor compared with other variables. Second, this study was conducted in the Asian population which obesity may be accounted for only 36.6% of patients with OSA^28^.

There are some limitations to this study. First, it was conducted in a single study site in a university hospital setting. Other aspects of OSA or other cardiovascular risk factors were not determined^29-32^. Second, there is no intervention in this study such as continuous positive airway pressure (CPAP) machine therapy^33-35^. Further studies are encouraged to evaluate the effects of CPAP therapy and AF reduction in OSA patients. Finally, further studies may be required to evaluate thromboembolism events in this setting. A previous study found that untreated OSA patients may have a poor response on catheter ablation of AF with a higher rate of recurrence^1^.

## CONCLUSION

The prevalence of persistent AF in OSA was 15.57%. Elderly patients with renal deterioration increased the risk of AF but AF risk was decreasing in patients with tiredness.
